# Hospital and Institutional News

**Published:** 1913-02-08

**Authors:** 


### HOSPITAL AND INSTITUTIONAL NEWS.

### MR. TURNER, F.R.C.S., ON FREE CHOICE OF DOCTOR.

We publish this week a remarkable interview with Mr. E. B. Turner, F.R.C.S., on the fight for free choice of doctor which is now proceeding, owing to the risk to that right of selection which is being too evidently threatened. Mr. Turner convincingly shows the three points on which the fight centres, makes unmistakable the facts of the clerical work which have caused so much controversy, and sums up the campaign which the London Medical Committee is organising in the Metropolis. We say with confidence that no clearer exposition of the evils of panel servitude, and of the only way to remedy them, has been put forward in the press.

### "THE HOSPITAL'S" SPECIAL BOOK NUMBER.

We publish next week, on the 15th instant, a Special Book Number which will cover ground not hitherto traversed in the technical or lay newspapers. Primarily, it will summarise the literature of the past year as it affects institutions at home and abroad, the aspects of construction, equipment, and administration being equally dealt with. Such books are not easy of general access, and it is believed that special interest will be found in this unique summary of a widely dispersed and many-languaged literature. The best new and recent medical works will also receive ample notice, and we believe that The Hospital's Special Book Number of February 15 will fill a place never yet occupied in journalism. The price of the Special Number will be 1d.

### THE DOOM OF THE DAY-BOOK.

Mr. T. C. Winn, M.R.C.S., of South Hackney, has published a dilemma on one of the horns of which he has succeeded in forcing the London Insurance Committee to impale themselves. "Finding it," he writes to the lay press, "utterly impossible to attend conscientiously to my patients and keep the records in the day-book provided by the London Insurance Committee, I wrote to them asking whether I should neglect my patients or drop the records." For once official circumlocution failed at the source, and in a dangerously terse letter the Clerk to the Committee, Mr. Thomas Batey, said "the day-book (Form Med. 24) is required to be kept by all medical practitioners" on the panel. This being impossible to the overworked doctor unless his patients are neglected, the inference that the day-book is more important than the diagnosis is naturally drawn by Mr. Winn. Perhaps the deaths of certain medical men on the panel have caused the Commissioners some compunction. At all events the Society of the Panel Doctors discussed simplifying the day-book in private with the Commissioners on Tuesday. If this means the ultimate doom of the day-book, as required at present, so much the better for all concerned.

### HOSPITAL FEES FROM INSURED PATIENTS.

The managers of Dr. Gray's Hospital, Elgin, Aberdeen, have achieved the unlucky distinction of having the legality of their recent resolutions with regard to insured persons questioned by the Elgin Town Council, which, by the way, under the founder's settlement, appear to have power to inspect the hospital. A resolution moved by Lord Provost Wilson was passed unanimously asking the managers, if the Town Council's legal advisers agree to such a course, to withdraw or amend their resolutions. These, it appears, contained clauses to the effect that "no insured person will be entitled to receive treatment at the hospital except in cases of emergency, unless an arrangement is made for payment to the managers of the hospital of £2 2s. per week as an indoor patient and £1 1s. per week as an outdoor patient." The Law Committee, to which the Council is entrusting its inquiry, consists of Mr. T. R. Mackenzie, convener, Bailie Black, Treasurer Shiach, Mr. Morrison, and Mr. Ramsay. The intention to charge insured persons some such fees for hospital treatment has been proclaimed by one or two, but only one or two, institutions. So far as we are aware the above is the first instance the legality of which has been impugned, and as such is worth attention.

### THE MANNERS OF LADY ALMONERS.

Although not the main question raised by Mr. G. A. Lewcock in a letter which he sends us this week concerning the recent complaints, noticed in our last issue, that have been brought against the Saturday Fund, his reference to the manners of lady almoners deserves notice not merely by the hospitals themselves and the Hospital Almoners' Council, but by all workers in the field of institutional after-care. "The main cause of our dissatisfaction," he writes, "is the way in which our subscribers are questioned and insulted by the so-called lady almoners at certain hospitals." That is a grave indictment to make, and hospitals and almoners alike will no doubt insist on the names of the institutions and their almoners which our correspondent has in mind being mentioned. So important is it in the best interests of the historic Saturday Fund to clear up the present discontent that we throw open our columns to any qualified correspondent who can check, amplify, or refute the view expressed in Mr. Lewcock's frank and therefore serviceable letter.

### "THE WORST INFIRMARY IN THE COUNTRY."

The Hartismere Board of Guardians, of which Mr. H. Nunn is chairman, has received a drastic letter from the Local Government Board containing the following:—"In regard to the most discreditable condition of the Union Workhouse at Eye, they"—the Board—"consider it imperative that the Guardians should take immediate steps for the erection of a new infirmary. Unless the Board receive definite and satisfactory assurances that this will be done they will have to consider the necessity of direct intervention in order to remedy the existing conditions." A new infirmary has often been proposed, and its building often delayed, and we therefore congratulate the Rev. W. H. Denison, who moved an amendment, eventually carried, to another delaying motion, which lays down that "immediate steps" are to be taken to build a new institution. If every clergyman acted in this manner, and was known to be a stern opponent of procrastination, it would increase the credit both of the Church and of the various Boards. What the old institution is like in the words of an expert authority deserves to be placed on record. Dr. Fuller, the Senior Medical Officer of the Local Government Board, is reported at the meeting to have said: "I have visited every workhouse infirmary in England and Wales, and I have no hesitation in saying that the infirmary of Eye is, if not the worst, one of the worst in the country." A further remark of Dr. Fuller's deserves preservation. In answer to Mr. Rose, who objected to this description and referred to the Ipswich Workhouse wards where, as The Hospital pointed out, infirmary patients had to sleep on the floors, Dr. Fuller said: "I would rather sleep on the floor at the Ipswich Workhouse than in a bed at Eye Infirmary," East Anglia. A more crushing indictment can rarely have been made, and practical results from Mr. Denison's amendment will be watched for carefully.

### DEATH OF A HOSPITAL ARCHITECT.

Mr. W. J. Ancell, architect, died at his residence in Hampstead on the 20th ult. Though he will probably be best remembered by the large works carried out in London, such as the Trocadero, the Popular Café, and the Strand Palace Hotel, he will also be remembered by the extensions carried out at Golden Square Throat Hospital and the erection of the Isolation Hospital at Mogden. At the time of his death he was actively engaged on the island-site hotel near Piccadilly Circus, which will contain over 1,000 bedrooms, and this work will be carried on under the charge of his chief assistant, Mr. F. J. Wills. Probably nothing taxes the capacity of the institutional architect more than problems of adaptation or reconstruction. Indeed, the adaptation of old buildings, or the grafting on them of modern extensions, forms a division of architecture by itself. Mr. Ancell's work at Golden Square and Mogden is, therefore, in its own way truly as indicative of his powers as the larger buildings already mentioned, if not, from the expert point of view, even more so.

### THE SHEFFIELD REVIVAL OF HOSPITAL SUNDAY.

Special interest attaches this year to the returns of the Hospital Sunday Fund Movement in Sheffield, from the fact that they are the first to follow the new methods of organisation which were introduced during the past year. At the time of writing the last totals have not come in, but already a larger total has been received than was recorded in 1912. In that year the sum realised was £2,017, an increase of £200 over that subscribed in 1911, and at present £2,089 has come in, so that only £45 more are needed to beat the previous highest record, which was made in 1892. The new method was ushered in by a special committee representative of all churches and chapels in the city and district, and a large amount of work has fallen to the honorary secretaries, Mr. S. T. G. Smith, Vicar of Walkley, and to the Rev. E. H. Reynolds, who, we regret to add, died a few weeks ago. The details of the organisation were entrusted to a sub-committee, of which Mr. J. H. Freeborough is the chairman, and sermons in the places of worship were supplemented by the distribution of Red-Cross envelopes, which all members of congregations who were prevented from attending service on Hospital Sunday were invited to use for sending in their subscriptions. As an instance of the success which often attended the despatch of Red-Cross envelopes, we may note that St. Mary's Church realised over £11 by this means. St. Mark's, St. Augustine's, and the Parish Church also have sent in substantial additions over last year's totals. As Hospital Sunday began at a Church of England clergyman's suggestion, we are always glad to note the successes of the churches on this occasion, and its best guarantee is such active co-operation as this begins to indicate among the clergy of all denominations themselves.

### MENTAL PATIENTS IN POOR-LAW INFIRMARIES.

The problem of dealing with short-time mental patients has been pressing on the Darlington Board of Guardians for some time, and it has been decided to erect a separate building for their accommodation. The Clerk of the Board, Mr. C. H. Leach, recently reported a suggestion to build a mental ward at the east end of the new infirmary ward. The advantage of this plan is that it leaves the land immediately behind the workhouse untouched, so that extensions to the infirmary, if necessary, can be built there. It is a pity that more details were not forthcoming of the new ward, for short-time mental patients do not always receive that consideration which is their due and which, whether short-time or not, ought to be extended to all classes in a modern infirmary.

### CURIOUS DISCOVERY AT A MENTAL HOSPITAL.

A highly interesting discovery is reported to have been made on a part of the estate of Cane Hill Mental Hospital at Coulsdon. The appearance of a mole-hill having attracted the attention of the Medical Superintendent, Sir James Moody, to the possibility of a pocket of sand or gravel existing in the chalk upon which the estate lies, excavation was undertaken recently and a deposit of clean sand was found. The land at this spot is about 400 feet above the sea-level. In the course of the digging some bones were discovered, and their examination has apparently satisfied Sir James Moody that these are of great antiquity. He therefore consulted Mr. Henry Dewey, of the Geological Survey, and the authorities at the British Museum, and ascertained that the discovery consisted of remains of the head of a hippopotamus and two pieces of an ivory tusk, probably that of a mammoth. There are several considerable fragments of the head of the hippopotamus, which include portions of the jaw with teeth in position, the articulation of the jaw-bones, two of the larger teeth, and one of the vertebræ, and there are also a number of small parts of bone which so far it has not been possible to piece together. It is suggested that the remains should be preserved in the Horniman Museum.

### AN AERIAL POST BED.

Prince Christian of Schleswig-Holstein, President of the King Edward VII. Hospital at Windsor, has sent a letter to Mr. D. Lewis-Poole thanking him for the donation of £937 14s. 2d. to the hospital out of the fund realised by the aerial post, of which the Duke of Teck was trustee. The following is a copy of the inscription on the tablet which has been fixed over the "Coronation Aerial Post Bed" in the King Edward Ward of the hospital: "This bed was endowed by a donation from the fund realised in aid of charities by the 'Coronation first United Kingdom Aerial Post, A.D. 1911,' organised by Mr. D. Lewis-Poole and Captain Wyndham, by sanction of His Majesty's Post-master-General, for the conveyance of mails by aeroplane from London to Windsor, and from Windsor to London, in September, 1911, to commemorate the Coronation of their Majesties King George V. and Queen Mary."

### HOSPITAL CERTIFICATES FOR INSURED PERSONS.

As exception has very naturally been taken by resident medical officers of hospitals to giving the medical certificates on the "signing-on" forms of the approved societies, which, besides taking much time, require an expression of professional opinion, a recently expressed view of the National Health Insurance Commissioners on the subject, which has been forwarded to The Hospital, will be of interest to hospital managers. In reply to an inquiry on the subject, the Commissioners remark: "Before a society can make such payments (*i.e.*, payments to insured persons or their dependants) it has to receive a medical certificate or other sufficient evidence that the member is incapable of work. The Commissioners consider it probable that any society would accept as sufficient evidence a certificate signed by the secretary or other officer of the hospital that the member was an in-patient, and that it would not be necessary to obtain a certificate from a member of the professional staff of the hospital." If the approved societies accept this view, the additional work of the secretarial departments of the hospitals could be lessened, as our correspondent points out, by the societies issuing a special certificate for hospital patients which a lay officer might properly sign.

### IS MATERNITY BENEFIT ALREADY A DEAD-LETTER?

A serious example of the confusion which the Insurance Act is causing in every branch of the Voluntary and Public Health Services came to light last week, when the Billericay Guardians, Essex, decided to defer making their usual subscriptions to various local nursing funds. The reason given by the Board is the striking one that doctors and nurses have raised their fees considerably since maternity benefit has come into force. Mr. J. T. West stated that in the case of nurses their fees had risen from 7s. 6d. to 15s. If this be so, maternity benefit will go the way of so many other State subsidies of wages and rates, to increase, namely, the wealth of the person in no need of State assistance, and to leave the person whose need was the object of aid as badly off as before. The definite question, Is maternity benefit already a dead-letter? can be settled only by information from every district. Mr. J. T. West, at any rate, and the Billericay Guardians have done a valuable piece of work in asking this question in the most pointed possible way.

### INSURED PERSONS AND POOR-LAW INFIRMARIES.

As we expected, denunciations of the Insurance Act on the part of insured persons, whose vaunted right to free choice of doctor is being threatened by Insurance Committees, of doctors both on and off the panel, of hospitals which are being unnecessarily burdened with patients for which, as we explained in our second leading article last week, they were not designed, are now being echoed by Poor-Law infirmaries. "The Act," said Mr. R. H. Green, one of the Poplar Guardians, at a meeting last week, "which was passed with the view of preventing people going to the Poor Law, is having the reverse effect." Out of a list of fifteen persons entitled to sickness benefits which the Guardians had before them, ten went to the Guardians' sick institution, although no sickness benefit is to be made in respect of an inmate of a workhouse infirmary. Mrs. Scurr said that cases with which the panel doctors could not deal because they required nursing, and which the hospitals could not receive because, as in instances of influenza for example, they were designed for more serious cases, were being turned away to Poor-Law institutions. Such patients, however, in many cases are contributors to the National Insurance scheme, and, not being destitute, the ratepayers naturally do not bargain to support them in their institutions. The Poplar Guardians are seeking information from other Boards, all of which are being presented with the same problem. The West Derby (Liverpool) Board of Guardians, for example, have appointed a committee to consider the best way of obviating the admission to their institutions of all insured persons who are not cases of urgency.

### THE DOGMA OF MEDICAL INFALLIBILITY.

Mr. George Robinson, a farmer, of White House, Ainderby, was summoned at the North Riding Police Court, Northallerton, by the North Riding Education Committee, for neglecting to send his grandchild to school. Unexpectedly the defence raised the old question of medical infallibility. For the defence it was stated that the county officials knew that the child was delicate, and had actually received three certificates from medical men affirming that the child was suffering from tuberculosis, but that in spite of this they ordered her to be examined by the county medical officer. Captain Gardner, who conducted the defence, advised the parent not to send her, as he regarded the action of the county officials as high-handed in going behind the back of private medical men. The case was dismissed, and the County Council were ordered to pay one guinea costs. The question which arises from the case, however, is not so easily disposed of, and it is one which the medical and legal professions, to say nothing of the public, have a strong interest in solving. When doctors disagree whose advice shall be taken? In Court, for instance, medical men, as expert witnesses, do flatly contradict each other, with the result that the expert witness is gradually being recognised as so much a darkener of counsel that we get magistrates, like Mr. Plowden the other day, saying that whereever possible they must be dispensed with. In the present instance we have the county medical officer being held up by education authorities as an unimpeachable authority, the implication being that the guardian of the child in question could easily get three medical men to sign certificates of exemption, whether there were any true physical disability or not. That is a serious implication to cast on the medical profession, but so long as the public conspire to regard medicine as a form of magic or cure-mongering rather than a science, so long will the opinions of its professors be held to be untestable by the ordinary processes of reason. Where expert witnesses are needed they should be paid and appointed by the Courts alone. The popular dogma of medical infallibility threatens to bring the profession into contempt.

### DEATH OF THE PRESIDENT OF THE SOCIETY OF MEDICAL OFFICERS OF HEALTH.

The possibilities which are thrown open to an energetic man by the Public Health Service were seldom better used than by Dr. J. F. J. Sykes, who died last week in his sixtieth year. Nominally and officially Medical Officer of Health for St. Pancras, he was really a public health administrator with a wide grasp and a statesmanlike purview, in which every side of the improvement of public health in London had its place. The Howard Medal awarded to him in 1900 by the Royal Statistical Society is an apt acknowledgment of one side of his work. As President of the Society of Medical Officers of Health, and a member of many foreign associations, he was a leader and example to medical officers generally. Health and housing in their protean forms led him to contribute frequent interesting papers, and problems dear to him, such as the spread of disease in barrack schools, death certification, and hospitals for diphtheritic patients, were made the subject of many of these studies and reports. He had been Medical Officer of Health for St. Pancras for twenty-eight years, and was one of the founders of the St. Pancras School for Mothers.

### PROBLEMS OF WARD FURNITURE.

To Mr. Otto Beit's gift of £5,000 for homœopathic research, which we noticed last week, must now be added a further donation of £300, which he has presented, through Dr. Burford, for furnishing the Rylands Ward. The fund for furnishing the new Sir Henry Tyler Wing is now happily completed, £3,469 having been spent for this purpose. The total cost of the site, building, and equipment has proved to be £48,000, which has been raised during the past six years, a matter on which the board and Mr. E. A. Attwood deserve to be congratulated. No problems are more exacting than those concerning equipment, and more than one heavy expense has been incurred through such trivialities. The craze, say, for rounded corners, for in some instances it has amounted to that, has prohibited devices for keeping the beds from knocking the walls behind them; and a thoughtless adoption of rubber wheels on the beds themselves without regard to the floor material and the consequent methods, some of which are very destructive to rubber, of polishing them are two examples. Many would thank Mr. Attwood for a hint of any new tips that he has learnt in the course of ordering the equipment for the new wing.

### THE NEW TUBERCULOSIS REGULATIONS.

As previously foretold in our columns, the new regulations for the compulsory notification of tuberculosis came into force on Saturday last. Medical officers are required to conduct their inquiries in such a way as to cause as little annoyance as possible to tuberculosis patients and to their friends, and notification will not involve publicity. It will be remembered that the effect of the new regulations is to make compulsory other forms of the disease besides pulmonary tuberculosis, an extension which logic demanded and which was bound to be made sooner or later.

### RIVERS OF TUBERCULOUS MILK.

The milk supply has received as much criticism of late as its purifying has excited disputation among critics, the latest of whom, Sir James Crichton-Browne, characterised the supplies as rivers of tuberculous milk, when addressing the Sanitary Association last Saturday. In dealing with the Pure Milk Bill, he suggested certain precautions beyond the safeguards which prospective legislation offered. Among them was a suggestion for the establishment of bacteriological laboratories at the depôts of the great milk companies, where a daily microscopical examination of centrifugalised milk for tubercle could be undertaken and certified. By this means, he said, the companies at small cost would impose an efficient check on farmers and give a guarantee to their customers. If, however, precautions on such a large scale have any chance of being undertaken at all, surely they ought to be carried out before the milk reaches the depôts?

### THE LESSON OF THE HOVE POISON CASE.

The case which is still occupying the attention of the Coroner at Hove has served the purpose of drawing public attention to the dangerous nature of synthetic hypnotics, and it would not be surprising if, as an outcome of the inquiry, veronal is placed in the Schedule of Poisons at an early date. In the course of his evidence Dr. Willcox declared that the sale of veronal and similar drugs should be subjected to some restrictions, and nearly a year ago the Council of the Pharmaceutical Society passed a resolution recommending the Privy Council to place certain drugs of this character in the Poisons Schedule. If this were done the sale would be restricted to chemists, who would merely be required to label the scheduled drugs with the word "Poison" and the name and address of the seller. At the present time these drugs may be sold by anyone, even without a label of any kind, the result being that there is nothing to impress upon the public their dangerous character. It is interesting to add, in this connection, that the gentleman known as the Squire of Brixton, who died "by misadventure," according to the verdict at Tuesday's inquest, had apparently taken an overdose of this drug.

### THE TUBERCULOSIS DISPENSARY AT ST. THOMAS'S HOSPITAL.

The Borough Council of Lambeth has been extremely fortunate in obtaining the co-operation of the governors of St. Thomas's Hospital in the establishment of a complete independent tuberculosis dispensary correlated to the dispensaries in the borough. The Council will be responsible for and will maintain a central dispensary in Effra Road, Brixton, which will serve the patients in the southern portion of the straggling borough area. In the northern part bordering the Thames, where tuberculosis is extremely prevalent, the tuberculosis department at St. Thomas's Hospital will be at the disposal of all patients, whether insured under the National Insurance Act or not. The governors have undertaken to maintain the department out of their funds, and to pay the salary of a medical officer, who shall devote his whole time to the work. They have appointed Dr. R. C. Wingfield, B.A., M.D., B.Ch. (Oxon.), M.R.C.S., L.R.C.P., who is the Louis Jenner Research Scholar, to the post. He will have the aid of the consulting staff at the hospital whenever he requires it. The valuable work which has been carried on by the lady almoner during the past eight years will be associated with the dispensary. Indeed, it will be under the control of the lady almoner's department. Full inquiry will be made as to the position of the patients. The general bacteriological examinations will be carried out in the clinical laboratory of the hospital. The patients who hitherto have been under treatment by the general physicians of the hospital are now transferred to this department, special times having been fixed. It will be a branch dispensary in close connection with Lambeth, and it will carry out the service required for the "inner wards" of the Lambeth Borough, that is for practically the whole of the north of Lambeth. A feature of the work is a small ward for the examination of special cases. The full control of the department remains in the hands of the governors.

### THE DIFFICULTIES OF A VISITING CHAPLAIN.

The question whether a paid chaplain should or should not be appointed at Firvale Workhouse Infirmary, Sheffield, has been engaging the attention of the Sheffield Guardians during the past month. Last week a meeting was held, at which a long discussion took place on the proposal made by the Vicar of Sheffield that the voluntary services rendered hitherto by the Church of England's representatives, the Rev. C. Galleymore and Mr. Robert Holmes, be discontinued, and that the Guardians should appoint a paid chaplain at a salary of £100 a year. The late Archdeacon Eyre used to contribute £120 a year out of his own pocket, and the present Vicar of Sheffield is prepared to add another £100 per annum to the salary which he suggests the Guardians should pay. He also proposes that a Roman Catholic and a Nonconformist "instructor" should be appointed to minister to their respective adherents. It was decided in the end to ask the Vicar of Sheffield to reconsider his decision to end the present unofficial arrangement. The discussion, however, was more interesting than its result. The Chairman, Mr. G. T. W. Newsholme, said that Mr. Galleymore and Mr. Holmes had an impossible task, there being 486 members of the Church of England in the infirmary alone. The Rev. E. L. Day also pointed out that in the infirm wards of the hospital there were many enfeebled patients who badly needed a spiritual consoler and friend. "It is not a proper thing," he added, "to wait till they reach unconsciousness, and to pray them into heaven, as generally happens now." A man's whole time could profitably be spent in visiting the sick and dying. Mr. Newsholme argued for the necessity of a full-time Church of England chaplain according to the regulations of the Local Government Board. That is the law, he remarked, according to the Act of 1847. No one can explain this away, and we can well imagine the difficulties in the way of a visiting chaplain, like Mr. Galleymore, who already has a large working-class parish of his own.

### ANOTHER PANEL CONUNDRUM.

The difficult cases which the present chaotic state of "medical benefit" leads to are plentiful. Hard cases may make bad law, as the lawyers say; but bad regulations certainly lead to hard cases. Some of them have been alluded to briefly in recent issues of The Hospital. This week another has come under our notice; and probably it is of a type which will be a fairly common source of difficulty and friction. An insured person went to see a panel doctor on account of some enlarged glands in the neck. The panel doctor noted several decayed teeth, and advised (sensibly enough) that the glands were probably caused by them. He recommended the patient to see her dentist, which she did, paying the latter, of course, his usual fees. The dentist declared that at least eight teeth ought to be extracted, and that it would be best to do this under ether. The question then arises—Is the panel doctor under contract to perform this service? Or is it outside the terms of his bargain with the Insurance Committee? On the face of it, we should say that it is not part of the duties of a panel doctor to administer anæsthetics for dental operations upon insured persons; and in the particular case we are quoting he did not do so, but the patient engaged another medical man, not on the panel, to procure the required anæsthesia. Our view of the strict legal right or wrong of the matter may or may not be correct; but, at any rate, the facts of the case are as we have recorded. The question would never arise at all if the administration of anæsthetics, whether for major or minor surgery or for dental operations, were an extra, remunerated separately from panel service altogether. We advocated this as long ago as last November, but without avail. The regulation whereby a panel doctor must himself provide and pay his anæsthetist whenever he undertakes minor surgery on a panel patient is indefensible from every point of view, including that of economy. It can only result in the shirking of all surgery by panel practitioners, and in the consequent overburdening of the voluntary hospitals with work which the Insurance Act contemplates should be done as part of medical benefit. The sooner this absurd provision is repealed, the better for all concerned.

### THE LATEST ELIXIR OF LIFE.

When a man with a great reputation in the medical profession lowers himself by proclaiming the virtues of some method of treatment which is soon shown to be absolutely useless, he cannot expect ever again to have his startling announcements regarded with equally startling faith. Therefore, a prudent scepticism is necessary in view of Dr. Doyen's recent assertion that in "Mycolysine-solution colloidale phagogène polyvalente" he has discovered an elixir of life which will cure ninety-nine diseases, including cancer and consumption. Such, at least, is the statement with which he is credited in a leading illustrated journal, which devotes a page to the subject, and publishes the Paris surgeon's photograph at the head of it. The elixir of life may be all that Dr. Doyen says it is. Yet we cannot forget that eight or nine years ago the same surgeon announced the discovery of a serum which was held out to be a certain cure for cancer; and that in point of fact it was valueless for that or any other therapy. Dr. Doyen, as a surgeon, has a reputation for originality and dexterity which is European. As an inventor he is looked upon askance by every scientifically-minded person; and his latest "discovery" sounds, on the face of it, so incredible that it will need a careful and impartial investigation before it can be accepted at its author's estimate. The pity of it is that ignorant people will at once rush in where doctors fear to tread, and so the cancer serum fiasco may be repeated all over again.

### THE GROWING DEMAND FOR DISPENSERS.

The demand for dispensers, created by the transfer of the dispensing of medicines for insured persons from doctors to chemists, which was mentioned in The Hospital last week, is becoming more pronounced. This is especially so in the industrial centres of the North, where an unusually large proportion of the population is insured under the Act. Owing to heavy pressure of the new work, chemists find it difficult to cope with prescriptions as expeditiously as their customers would wish. If the work were evenly distributed throughout the day the difficulty would not be so great, but most of the prescriptions are brought in to the pharmacist after six o'clock in the evening, when the insured persons have finished work. A Manchester evening paper, in calling attention to the need for assistance, says "there is a big demand for dispensers and assistant dispensers"; while at a recent meeting of Manchester chemists a similar statement was made. As has been stated previously in The Hospital, any dispenser with practical experience is eligible to dispense medicines under the supervision of a pharmacist, and the salaries paid by pharmacists are not likely to be less than those paid to doctors' dispensers.

### PROPOSED TRADE UNION OF PHARMACISTS.

The new President of the Liverpool Chemists' Association, Mr. H. Humphreys Jones, took advantage of the annual meeting last week to deliver an instructive address on the position of the pharmacist under the Insurance Act. After warmly congratulating Mr. Glyn-Jones, the Parliamentary Secretary, on the amendments that his activities had secured, the President said that these were so far-reaching as probably to revolutionise pharmacy, and that Mr. Lloyd George would be remembered, willingly or unwillingly, as the first statesman to recognise the claims of pharmacists. Immediate results from the Act might prove disappointing to them, but greater issues lay dormant. They had the Chancellor's pledge to readjust their tariff if at the end of the experimental three months the scale was found to afford an insufficient margin of profit. "The time has now arrived," he concluded, "when a strong national movement in the nature of a trade union of pharmacists should be formed to regulate the practice and forward the interests of its members." He appealed for support for such a society which had already been formulated by a member of their Association. Interest in such a proposal must extend far beyond Liverpool, and we invite the criticisms and suggestions of pharmacists upon the project.

### THIS WEEK'S DRUG MARKET.

Business continues to be fairly good, but the demand for drugs which are usually in common use at this time of the year is comparatively small, owing to the unseasonable weather. Changes in value of any note have been few since last report appeared. Essence of lemon continues to advance. Cocaine is 7d. per ounce lower. Atropine salts are slightly dearer. Reports from Norway are to the effect that the cod-fishing has begun under favourable conditions, but it is too early as yet to form any opinion of what the results are likely to be. Cod-liver oil is one of the products the demand for which has suffered in consequence of the mild season; on the other hand, this drug has been more largely prescribed for consumption patients as part of the sanatorium benefit under the Insurance Act. Opium is without change. There has been no further alteration in the position of quinine. Orris-root continues to show an advancing tendency. Cream of tartar is slightly dearer.

